# A pathological indicator for dysthyroid optic neuropathy: tritan color vision deficiency

**DOI:** 10.1007/s00417-021-05227-8

**Published:** 2021-06-22

**Authors:** Aylin Garip Kuebler, Kathrin Halfter, Lukas Reznicek, Annemarie Klingenstein, Siegfried Priglinger, Günther Rudolph, Christoph Hintschich

**Affiliations:** 1 Department of Ophthalmology, Ludwig-Maximilians-University, Mathildenstr. 8 80336, Munich, Germany; 2 The Institute for Medical Information Processing, Ludwig-Maximilians-University, Biometry and Epidemiology Marchioninistr. 15, 81377, Munich, Germany

**Keywords:** Dysthyroid optic neuropathy, Tritan deficiency, Protan deficiency, Color vision test

## Abstract

**Purpose:**

To investigate the sensitivity of the color vision test by Arden in patients with dysthyroid optic neuropathy (DON) to improve diagnosis.

**Methods:**

In this observational, retrospective study, we included the medical records of 92 eyes (48 patients) with diagnosis of DON between 2008 and 2019 in order to evaluate the full spectrum of findings from the color vision test by Arden, and to determine potential importance of this test. Thirty-five patients were female, and 13 patients were male. The mean age was 58.0 years (range: 34–79) at the time of the DON diagnosis.

**Results:**

Forty-one eyes displayed relatively good BCVA with ≤ 0.2 LogMAR. We found a protan value exceeding the threshold of ≥ 8% in 57 eyes (30 patients) at the time of the diagnosis. The sensitivity of protan was 61.9% (95% CI 51.2–71.8%), while that of tritan was a striking 98.9% (95% CI 94.1–99.9%).

We discovered one pathological sign, tritan deficiency (based on a threshold of ≥ 8%) consistently in all eyes but one at the time of the diagnosis, regardless of the visual field defects or any changes in best-corrected visual acuity (BCVA).

**Conclusion:**

We found blue-yellow (tritan) deficiency, to be a sensitive and reliable indicator of dysthyroid optic neuropathy. We conclude that, in cases with suspected DON, a color vision test that can detect tritan deficiency is an essential tool for the adequate assessment, diagnosis, and treatment of DON.


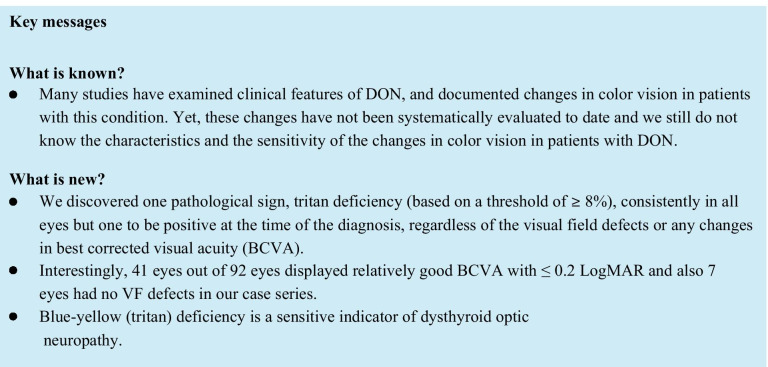


## Introduction

Thyroid-associated orbitopathy (TAO) is the most common manifestation of Graves’ disease, affecting 25–50% of patients with the condition [1]. TAO presents as an orbital inflammation leading to an enlargement of the extraocular muscles and proliferation of the orbital fat, which, in turn, result in exophthalmos, impairment of the ocular motility, eye lid retraction, and, in severe cases, compression of the optic nerve. About 40% of the patients with Graves’ disease suffer from a simultaneous manifestation of ocular and systemic symptoms [2, 3].

Dysthyroid optic neuropathy (DON) is another serious complication of TAO, observed in 4–8% of the patients [4, 5]. Although considered self-limiting, with an active phase lasting up to 24 months, followed by an inactive phase, DON still needs to be diagnosed in a timely manner to prevent the vision loss.

According to the Consensus Statement of the European Group on Graves’ Orbitopathy (EUGOGO), DON is described as sight-threatening TAO and/or corneal breakdown requiring immediate intervention to preserve vision [6]. In most cases, the diagnosis of DON is quite challenging due to the multifactorial causes of visual acuity impairment in TAO patients.

This study seeks to identify the potential pathological signs of DON by analyzing the full spectrum of findings from color vision tests in a large group of patients. The ultimate goal is to aid early diagnosis of DON and to allow for its timely treatment.

## Patients and methods

### Patients

This observational, retrospective, cross-sectional study was conducted in the Department of Ophthalmology, Ludwig-Maximilians-University, Munich, Germany. For this type of study, formal consent is not required. All procedures performed in this study were in accordance with the ethical standards of the institutional research committee and with the Helsinki declaration and its later amendments or comparable ethical standards. We obtained approval from the Ethics Committee of the Ludwig-Maximilians-University, Munich, Germany.

We examined the medical charts of all patients given a definitive diagnosis of DON in the Plastic and Reconstructive Surgery Section between 2008 and 2019. Our sample included 48 patients and 92 eyes. We compiled and analyzed the data with SPSS version 25.0 (SPSS Inc., Chicago, IL, USA).

The definitive diagnosis of DON was based on the presence of one or more of the following clinical findings, confirmed by a senior ophthalmic plastic consultant (CH): (i) worsening of best-corrected visual acuity (BCVA) in comparison to the prior visit (≥ 2 lines) and/or (ii) BCVA with pinhole being ≤ 0.6 (decimal), but not due to corneal problems or other pre-existing eye diseases; (iii) a positive swinging-flash-light test or relative afferent pupillary defect (RAPD); (iv) optic disc edema; or (v) findings of apical crowding in orbital computerized tomography (CT).

We included only the patients with an existing color vision test by Arden at the time of the diagnosis of DON, which allowed us to evaluate patterns of protan and tritan deficiency in DON patients.

Exclusion criteria were corneal pathologies including significant keratopathy, which potentially affect the outcome of the color vision test; history of corneal or glaucoma surgery; nystagmus; other causes of optic neuropathy; and coexisting ocular diseases such as amblyopia, retinal pathologies, and significant cataract as documented in the medical records.

In all patients, a complete ophthalmic examination was performed including BCVA assessed in decimals, obtained with a Snellen projection chart at a distance of 6 m, and documented as LogMAR function for statistical analysis. In addition, slit-lamp biomicroscopy, ocular motility, measurement of proptosis with Hertel exophthalmometry, relative afferent pupillary defect or swinging-flash-light test (SWFLT), and indirect ophthalmoscopy were recorded. Assessment of the activity of Graves’ orbitopathy (GO) was done according to the European Group on Graves’ Orbitopathy (EUGOGO) recommendations with the clinical activity score (CAS) including 7 items at the first visit and 10 items at the following visits (ocular or retrobulbar pain, pain with eye movement, erythema of the eye lids, swelling of the eye lids, redness of the conjunctiva, chemosis of the conjunctiva, swelling/erythema of caruncle, increase in proptosis (≥ 2 mm), worsening of extraocular muscle motility in any one direction (> 8°), and/or worsening of visual acuity (≥ 1 line decrease in Snellen chart) [7].

### Color vision test by Arden

In all patients, a computerized color vision test by Arden was performed. In this examination, a 21-inch. color monitor was used to present 10 random letters (A, E, H, M, O, T, U, V, X, and Y) for 200 ms in the middle of the monitor to patients from a distance of 1.5 m. The letters have an equal luminance with the background and can only be recognized due to their difference in hue from the background. In this method, three color axes—red (protan), green (deutan), and blue (tritan)—can be tested. The color vision test self-calibrating is also additionally being calibrated and checked every 6 months by a professional. After the test, a modified binary search is carried out to identify the threshold color contrast. According to Berninger et al., the threshold for the tritan and protan axes should not exceed 8% in healthy probands [8]. According to their work, protan and deutan results were comparable; therefore, only the data for protan and tritan was collected. Accordingly, in our study, we considered protan and tritan to be pathological if the tested threshold was ≥ 8.0%.

Arden color vision test was described by Arden et al. in 1988 [9, 10]. At their first published preliminary results, the authors described a computer graphics system, which calculates the values of red, green, and blue signals and specifies them in Commitè Internationale d’ Eclairage (CIE) X, Y, and Z fundamentals, so the changes in chrominance happen without any changes in luminance. Also, the authors argued that this method has the main advantage of patients being presented with graduated and constantly changing hues in which no alterations of luminance occur. The other advantages are this method being self-calibrating and automated, avoiding operator error and also the difficulties of interpretation.

Also, it is important to mention that there is a possible effect of aging on color vision. As an example, Barbur et al. showed that thresholds for pathology increase about 1% per year for red-green and about 1.6% for blue-yellow past the age of 20 years [11]. However, there are two other researches arguing that there are no age-driven pattern in color vision thresholds [8, 12]. Further prospective studies with a larger number of patients are needed for a better understanding of the changes in color vision with aging.

## Results

We included 48 patients, 92 eyes in the study. Thirty-five patients were female and 13 patients were male, with a mean age of 58.0 years (range 34–79).

Forty-three patients (89.6% of all cases) had a bilateral involvement at the time of DON diagnosis, and the remaining 6 patients (10.4% of all cases) had unilateral involvement. Five patients had a diagnosis of asthma, 8 patients had diabetes, and 9 patients had high blood pressure as accompanying conditions to Graves’ disease.

Nineteen patients were current-smokers, 9 patients were ex-smokers, and 17 had no smoking history (*n* = 45 patients). The fT3 was 2.88 µg/dl on average (range 0.90–5.80, *n* = 35), fT4 was 2.98 µg/dl (range 0.50–22.80, *n* = 35), and thyroid-stimulating hormone (TSH) was 3.67 μU/ml (range 0–34.00, *n* = 43). The mean TSH-receptor antibody (TRAB) was 19.40 mIU/ml (range 0.71–110.00, *n* = 43); the mean intraocular pressure (IOP) was 18.5 mmHg (range 12–32 mmHg, *n* = 91 eyes) at the time of the DON diagnosis.

A total of 84 eyes had visual field defects compatible with DON, while 7 eyes had no VF defects at the time of the diagnosis; due to the retrospective design of the study, the VF of one eye was missing. The mean deviation (MD) of the VF was − 8.1 on average (range − 32.9 to 1.3, *n* = 91 eyes). The mean PSD was 4.88 (range 0.60–11.40, *n* = 91 eyes).

The summary of the demographic profiles and clinical findings can be seen in Table 1.

**Table 1:** Demographic data for all included patients and clinical data for all included eyes

Characteristic	*n* (%)/mean (range)
All	48 (92 eyes)
**Patient demographic data**	
Age	58.0 (34–79) years
Sex	
Male	13 (27.1%)
Female	35 (72.9%)
Smoking history	
Current	19 (39.6%)
Ex-smoker	9 (18.8%)
None	17 (35.4%)
Missing	3 (6.3%)
TRAB	19.4 (0.7–110.0)
TSH (µU/mL)	3.7 (0–34.0)
**Clinical data for all included eyes**	
BCVA (LogMAR)	0.42 (0–2.0)
CAS	5.6 (1–8)
Visual field mean deviation (dB)	− 8.1 (− 32.9 to 1.3)
Hertel exophthalmometer (mm)	21.7 (14–28)
RAPD	
Negative	76 (82.6)
Positive	16 (17.4)
Optic disc edema	
Yes	26 (28.3)
No	66 (71.7)
Visual field defect	
Yes	84 (91.3)
No	7 (7.6)
Missing	1 (1.1)
Protan	27.7% (1.4–100.0)
≥ 8.0%	57 (30 patients)
< 8.0%	35 (18 patients)
Sensitivity	61.9% (95% CI [51.2–71.8%])
Tritan	41.6% (6.3–100.0)
≥ 8.0%	91 (48 patients)
< 8.0%	1
Sensitivity	98.9% (95% CI [94.1–99.9%])

*TRAB* TSH-receptor antibody, *TSH* thyroid-stimulating hormone, *BCVA* best-corrected visual acuity, *CAS* clinical activity score, *RAPD* relative afferent pupillary defect

At the time of the DON diagnosis, 26 eyes (12 patients) had an optic disc edema, and 10 patients (16 eyes) had a positive swinging-flash-light test.

The mean BCVA was 0.42 LogMAR (range 0–2.0), with 22 patients (41 eyes) displaying a BCVA ≤ 0.2 LogMAR.

The mean clinical activity score (CAS) was 5.6 (range 1–8, *n* = 92 eyes). The mean protan value was 27.7% (range 1.4–100%, *n* = 92 eyes), with 57 eyes (30 patients) showing a pathological protan value exceeding the threshold of ≥ 8%.

The most striking finding came from the tritan values (mean = 41.6%, range 6.3–100%, *n* = 91). All 48 patients and 91 eyes, except for one eye, displayed the same pathological sign of tritan deficiency (exceeding the accepted threshold of 8%) at the time of the DON diagnosis. There was only one eye with a normal tritan value of 6.3%. This sign was observed regardless of the presence of VF defects or any changes in BCVA. Indeed, 41 eyes had a relatively good BCVA with ≤ 0.2 LogMAR. While BCVA (LogMAR) values certainly correlated with tritan and protan values at a statistically significant level (both *p* < 0.0001, Pearson correlation coefficient), that correlation was far from perfect. Thus, tritan was able to point to a pattern that was undetectable with BCVA or protan alone.

We also calculated the sensitivity of the protan and tritan test, and 95% confidence interval was calculated using the Clopper-Pearson method. While the sensitivity of protan remained at 61.9% (95% CI: 51.2–71.8%), that of tritan was a remarkable 98.9% (95% CI 94.1–99.9%).

## Discussion

The diagnosis of the dysthyroid optic neuropathy (DON) in thyroid-associated orbitopathy (TAO) is based on many clinical features including decreased BCVA, changes in color vision, relative afferent pupillary defect (RAPD), optic disc edema, VF defects, apical crowding on CT images, and reduced amplitude on visual-evoked potentials [13, 14]. Lack of standardized diagnostic criteria is a major impediment to timely diagnosis and treatment of DON. Existing diagnostic tools, such as visual field (VF) examination, color vision, and visual acuity tests, not only require patient cooperation, but also are largely subjective and easily influenced by coexisting corneal problems and motility of the eye (e.g., double vision) seen in TAO patients. The diagnosis of DON is especially challenging for patients who display no or minimal change in BCVA and VF. This study evaluated color vision tests as an alternative tool for identifying neuro-ophthalmic pathology, which can be missed in tests for visual acuity or visual field.

Many studies have examined clinical features of DON, and documented changes in color vision in patients with this condition. Yet, these changes have not been systematically evaluated to date. Our study analyzes the full spectrum of findings from color vision test by Arden in a large sample of patients (*n* = 48 patients, 92 eyes) diagnosed with DON. We focus on a particular modality, computerized color test by Arden, which allows for precise and fast detection of individual color vision along the protan and tritan axes.

A number of studies have noted the importance of color vision deficiency in the detection of DON [5, 12, 15]. In McKeag et al.’s study for example, 80% of the patients with definite DON displayed reduced color vision as opposed to 56% of the patients with equivocal DON and 7% of the patients without any clinical signs of the condition. Based on this observation, the authors concluded that impaired color vision, along with optic disc swelling and radiological evidence of apical optic nerve compression, can be a useful parameter in confirming DON diagnosis [15].

Neigel et al. investigated clinical features of DON with a large case series of 58 patients (95 eyes) diagnosed with the condition. According to their results, the authors found the Farnsworth-Munsell 100-hue test to be a sensitive indicator for color discrimination in DON patients. Yet, the authors also used pseudoisochromatic screening procedures (Ishihara and Dvorine plates) and found that those procedures rarely identified the acquired color defects in DON patients unless optic neuropathy was severe. According to the authors, the most sensitive indicators of optic nerve dysfunction in DON patients appeared to be visual-evoked potentials and color vision. Interestingly, the results nevertheless showed a main symptom of differentiated patients with DON was graying of vision, or desaturation of colors in optic neuropathy patients. The patients rarely voluntarily reported this symptom unless specifically asked [5].

Our results, based on 48 patients and 92 eyes, are generally in line with McKeag et al. and Neigel et al. in pointing to color deficiency as a sign of DON. Our findings, however, are more specific in identifying the particular forms of color deficiency in DON patients compared to earlier reports. We observed a pathological protan value (exceeding the threshold of 8%) in 30 patients (57 eyes) at the time of DON diagnosis. Remarkably, we discovered a pathological tritan deficiency (based on the same threshold of 8%) in all eyes except for one eye (91 eyes of the 48 patients). Put differently, while the sensitivity of protan measure was 61.9% (95% CI 51.2–71.8%), that of tritan was an impressive 98.9% (95% CI 94.1–99.9%).

Today Ishihara plates remain the most popular method for testing color vision deficiency. The method is simple, easy to use, time sparing, and useful for the screening red-green congenital deficiency. Yet the method is not ideal for testing acquired color deficiency since it cannot quantify the severity, or identify the blue-yellow deficiency [16, 17].

Our results might be relevant to a broader area of diseases which cause changes in color vision especially tritan deficiency such as optic neuritis, macular diseases, media opacity, dominantly inherited juvenile optic atrophy (DIJOA), and amblyopia [18, 19]. It is important to mention that there are a broad spectrum of hereditary diseases strongly related with blue-yellow deficiency such as DIJOA and congenital tritanopia [19].

For example, Almog et al. documented color vision loss as an outcome of optic neuropathy even among patients with a good-to-moderate visual acuity (VA). This finding is all the more striking given that the authors rely on Ishihara plates for testing color vision, which is limited in identifying acquired color deficiency. Although the focus of our study detailed above was on a different condition (DON) and relied on a different color vision test (by Arden), we observed a similarly striking pattern. Ninety-one eyes (out of 92 eyes) in our data display pathological values of tritan, even the 41 eyes that had a relatively good BCVA of ≤ 0.2 LogMAR.

We conclude that tritan deficiency may indicate the optic nerve compression in patients with DON. Research suggests tritan deficiency to be one of the most common forms of acquired vision loss. Jafarzadehpur et al., for instance, observed tritan deficiency in 66.1% of their cases with acquired color vision loss. Similarly, tritan (blue-yellow) deficiency is linked to retinal diseases in one of the earliest classification of color vision loss, dating back to 1912, while protan (red-green) deficiency is associated with optic nerve pathology [20]. Current research recognizes many exceptions to this generalization, also known as the Köllner’s rule, and is particularly suspicious of its applicability to optic neuropathies, such as optic neuritis [21].

Today, the Verriest classification—based on a retrospective analysis of 544 eyes—is the gold-standard classification for acquired vision deficiencies [22]. In this scheme, tritan (blue-yellow) deficiency (defined as Verriest type III color vision deficiency) is linked to vascular retinopathies, papilledema, glaucoma, and dominant optic atrophy, but not yet to DON.

Our study presents evidence for the potential importance of tritan deficiency in the diagnosis of DON. Most importantly, we showed that tritan deficiency can help detect DON even in cases with little vision loss. A total of 17 DON patients (41 eyes) in our data had a relatively good BCVA score of ≤ 0.2 LogMAR, but they all showed pathological tritan values. Indeed, the sensitivity of tritan was 98.9% in our study (compared to only 61.9% of protan). Clinical diagnostics could use tritan tests to confirm a suspected DON diagnosis, and also to potentially detect patients early before they display any signs of vision loss.

Our sample is relatively large (*n* = 48 patients, 92 eyes) considering the rarity of the disease yet a larger sample size and prospective study design would be useful in confirming our findings. Our method of choice (Arden color vision test), although being not a well-known test method for color vision, is not the only means by which to detect tritan deficiency. A limitation of our research was not having a control group due to the retrospective design of the study. Future work could evaluate alternative tests for blue-yellow deficiency in detecting optic nerve compression DON patients. Regardless of the method, we argue that a color vision test that can detect tritan deficiencies may be an essential and sensitive tool for the adequate assessment, diagnosis, and treatment of DON.
